# Minimally Invasive Parathyroidectomy in a Child With Acute Pancreatitis

**DOI:** 10.7759/cureus.24058

**Published:** 2022-04-12

**Authors:** Fiaz Ali, Panduranga Seetahal-Maraj, Lakhan Roop, Rae-Ann Mohammed, Vijay Naraynsingh

**Affiliations:** 1 Paediatric Surgery, San Fernando Teaching Hospital, San Fernando, TTO; 2 Neurosurgery, San Fernando General Hospital, San Fernando, TTO; 3 Paediatrics and Child Health, San Fernando Teaching Hospital, San Fernando, TTO; 4 Clinical Surgical Sciences, University of the West Indies, St. Augustine, TTO; 5 Surgery, Medical Associates Hospital, St. Joseph, TTO

**Keywords:** minimally invasive parathyroidectomy, hypercalcaemia, parathyroid adenoma, hyperparathyroidism, paediatric, acute pancreatitis

## Abstract

Acute pancreatitis (AP) is rarely seen in the paediatric population and is typically not associated with those aetiologies seen in adult pancreatitis. This case describes a 12-year-old female who presented with acute abdominal pain and constipation, with biochemical evidence of elevated serum amylase, calcium (Ca) and parathyroid hormone (PTH) levels. A diagnosis of AP was made, which was settled with conservative management. Further investigations, namely CT and technetium 99m (Tc-99m) sestamibi scans, revealed a solitary parathyroid adenoma. She subsequently underwent minimally invasive parathyroidectomy (MIP), following which Ca and PTH levels normalized postoperatively.

## Introduction

The typical causes of acute pancreatitis (AP) in adults are not the usual culprits in the paediatric population. We present the case of a 12-year-old girl with AP secondary to hypercalcaemia. Further investigations revealed primary hyperparathyroidism (PHPT) due to a solitary parathyroid adenoma. The patient underwent successful minimally invasive parathyroidectomy (MIP), after resolution of acute pancreatitis. On three-year follow-up, the patient remains symptom-free, with normal plasma Ca levels.

## Case presentation

A 12-year-old female with a four-day history of generalized abdominal pain and constipation was referred to our unit. The pain was constant, maximal in the epigastrium, without radiation and showed mild relief on leaning forward. Additionally, movement and oral intake aggravated the abdominal pain. The patient reported a three-week history of polyuria and polydipsia, but denied any history of musculoskeletal pain, psychiatric symptoms or renal colic. Previous surgical history revealed an uncomplicated open appendicectomy at eight years of age. Further enquiry indicated no prior medical history, medication usage or any known family history of comorbidities.

Examination revealed tachycardia with a pulse of 124 beats per minute, but no derangement in blood pressure and normal mucous membranes. An abdominal examination demonstrated a soft abdomen, with notable guarding in the epigastric region but no flank tenderness, peritonitis or abdominal wall bruising. Rectal and cardiopulmonary examinations were normal.

Abdominal X-ray displayed faecal loading. Laboratory investigations showed an elevated serum amylase of 1400 U/L, serum Ca of 18 mg/dl and alkaline phosphatase (ALP) of 1849 IU/L. PTH levels were found to be 1244 pg/ml (the normal range is 15-68 pg/ml). Renal function tests were within normal limits. CT scans of the abdomen and pelvis revealed findings suggestive of acute pancreatitis (Balthazar C), diffuse bony sclerosis and incidental bilateral non-obstructing renal calculi at the lower poles.

The patient was kept fast and placed on appropriate analgesia and intravenous fluids. Loop diuretics and intravenous fluids facilitated normalization of the serum calcium to 9.1 mg/dl. Over five days, the attack of acute pancreatitis resolved with conservative management.

**Figure 1:**
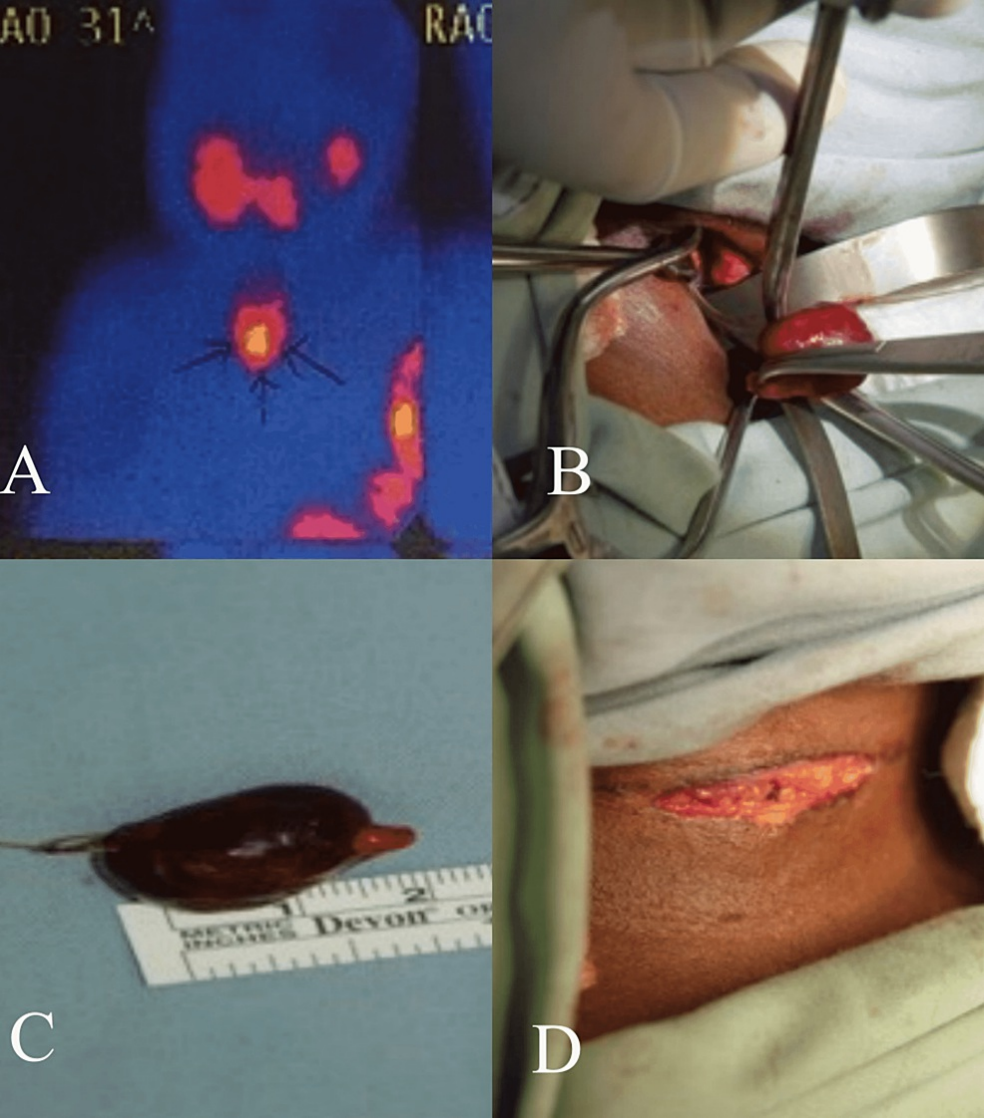
Parathyroid adenoma (A) Tc-99m sestamibi scan showing increased uptake in the right inferior pole of the thyroid. (B) Adenoma delivered from a wound. (C) Excised parathyroid adenoma. (D) Closure of the minimally invasive incision. Tc-99m, technetium 99m.

CT scan of the neck showed a 1.7 cm x 1.2 cm well-circumscribed mass in the right retrothyroid region, suggestive of a parathyroid adenoma. Technetium 99m (Tc-99m) sestamibi scan revealed an area of increased uptake overlying the right lower pole of the thyroid gland (Figure 1A).

Ultrasound scanning preoperatively localized the lesion and allowed skin marking to aid in minimal dissection at the surgery. The patient then underwent a minimally invasive right inferior parathyroidectomy (Figure 1B-D). On gross examination, the adenoma was pink and rubbery and measured 2.5 cm x 2.5 cm x 2.0 cm, with a weight of 3.9 g. Following this excision, serum PTH levels fell from 1244 pg/ml preoperatively to 119 pg/ml intraoperatively, confirming a successful resection. Serum Ca fell from 12.6 mg/dl preoperatively to 7.7 mg/dl within 48 h post-excision. The patient spent an uneventful postoperative period, was subsequently discharged and is currently followed up in the Paediatric Surgery outpatient clinic for monitoring of serum Ca and PTH levels.

Six weeks after initial presentation, an abdominal ultrasound revealed the development of a 4 cm x 4 cm x 5 cm pancreatic pseudocyst, with no compressive symptoms. Conservative management was employed in this instance and there was complete resolution within three months of diagnosis. The renal calculi seen on the previous CT scan also resolved spontaneously. Final histology confirmed a parathyroid adenoma with no features of malignancy. On three-year follow-up, the patient continues to be symptom-free, with normal serum Ca levels.

## Discussion

While the leading causes of AP in adults include gallstones and alcohol (>60%), in children the major cause is abdominal trauma (23-40%). Pancreaticobiliary anomalies (15%), systemic disease (14%), drugs and toxins (12%), viral infections (10%), hereditary disorders (2%) and metabolic disorders including hypercalcaemia (2%) account for the rest [1,2].

The diagnosis of AP in the paediatric population does not readily come to mind as a differential for acute abdominal pain. Due to under-reporting or non-diagnosis, the true incidence is unknown. Currently, only limited data is available on the incidence of paediatric AP in the Caribbean region and there are no reported cases.

PHPT in children has an incidence of 2-5 per 100,000, of which the most common cause is a solitary adenoma [3-6]. The aetiology of PHPT due to a single adenoma is associated with somatic mutation in MENIN, PRAD1 and HPRT2 (CDC 73) genes [7].

While PHPT has been reported in adolescents, it is rarely found in the under-10-year-old age group. Despite the lower incidence, symptoms of PHPT are more common and more severe in children and adolescents when compared to adults. 70-90% of adolescent patients with PHPT would present with symptoms as opposed to their adult counterparts, who would be symptomatic on presentation 20-50% of the time.

Despite the high frequency of symptoms in children with PHPT, definitive diagnosis is often significantly delayed [8]. This is due to several factors including infrequent laboratory screening tests, the wide range of ALP levels in children and the myriad of vague, non-specific presenting symptoms such as abdominal pain, bone pain, nausea, vomiting, headache and so on. Hence, making the diagnosis of PHPT on time is often challenging and has been reported to range from as long as seven months, to more than four years [9].

In the routine investigation of abdominal pain, serum amylase should be analysed. As in our patient, the presence of abdominal pain and elevated amylase (more than three times the upper limit) aided in further investigation of the aetiology. Electrolytes were checked and the underlying cause of the AP was then found to be hypercalcaemia.

The combined presentation of AP and renal calculi adds to the uncommon nature of this case. However, the presence of the renal calculi hastened the search for the source of elevated Ca levels. Imaging studies revealed a solitary adenoma on Tc-99m sestamibi scanning and a likely diagnosis of PHPT. Metabolic imaging with Tc-99m sestamibi scintigraphy allowed precise localization and has a reported sensitivity of 86% in children. Ultrasound is also reported to have a good sensitivity of 79% [9]. MRI evaluation can also be used as it is highly sensitive (88%), but is restricted to difficult cases with ectopic or multiple adenomas, or after failed localization with ultrasound or sestamibi scans [9].

Surgical management of PHPT in children due to a single adenoma can be via MIP. This technique is dependent on successful preoperative localization of a single adenoma by Tc-99m sestamibi scan or ultrasound [10]. This method offers numerous advantages such as less dissection, improved cosmesis and a lower risk of neurovascular injury. Intraoperative PTH assay is done to confirm a reduction in PTH of greater than 50% and thus successful surgery.

Postoperatively, this patient spent an uneventful course, with transient hoarseness from endotracheal intubation being the only acute complication. PTH levels fell by 90.43% intraoperatively and serum Ca levels fell by 38.88% postoperatively. Studies have shown that a decrease in intraoperative PTH levels of 80% or more is a risk factor for the development of postoperative hungry bone syndrome (HBS) [11]. However, our patient had stable serum Ca levels and did not develop HBS postoperatively. Nevertheless, prophylactic oral Ca supplementation was given.

## Conclusions

In this uncommon case of paediatric PHPT presenting as AP with renal calculi, the need to be mindful of this diagnosis is highlighted. Children do not have the typical aetiologies of AP seen in adults, and investigations should be tailored to this fact. Successful MIP was done with the help of adequate preoperative localization. Despite the drastic decline in PTH levels following the successful removal of the adenoma, our patient did not develop HBS. However, a high index of suspicion should be maintained, as this is a well-documented complication of parathyroid surgery. On three-year follow-up, the patient remains asymptomatic and clinically well.

## References

[1] Morinville VD, Husain SZ, Bai H (2012). Definitions of pediatric pancreatitis and survey of present clinical practices. J Pediatr Gastroenterol Nutr.

[2] Suzuki M, Sai JK, Shimizu T (2014). Acute pancreatitis in children and adolescents. World J Gastrointest Pathophysiol.

[3] Venail F, Nicollas R, Morin D (2007). Solitary parathyroid adenoma: a rare cause of primary hyperparathyroidism in children. Laryngoscope.

[4] George J, Acharya SV, Bandgar TR, Menon PS, Shah NS (2010). Primary hyperparathyroidism in children and adolescents. Indian J Pediatr.

[5] Mallet E (2008). Primary hyperparathyroidism in neonates and childhood. The French experience (1984-2004). Horm Res.

[6] Hsu SC, Levine MA (2002). Primary hyperparathyroidism in children and adolescents: the Johns Hopkins Children's Center experience 1984-2001. J Bone Miner Res.

[7] Roizen J, Levine MA (2012). Primary hyperparathyroidism in children and adolescents. J Chin Med Assoc.

[8] Kollars J, Zarroug AE, van Heerden J (2005). Primary hyperparathyroidism in pediatric patients. Pediatrics.

[9] Bauman BD, Evasovich M, Louiselle A (2017). An occult ectopic parathyroid adenoma in a pediatric patient: a case report and management algorithm. J Pediatr Endocrinol Metab.

[10] Belcher R, Metrailer AM, Bodenner DL, Stack BC Jr (2013). Characterization of hyperparathyroidism in youth and adolescents: a literature review. Int J Pediatr Otorhinolaryngol.

[11] Ebina K, Miyoshi Y, Izumi S (2015). A case of adolescent giant parathyroid adenoma presenting multiple osteolytic fractures and postoperative hungry bone syndrome. Clin Case Rep.

